# The German Shorthair Pointer Dog Breed (*Canis lupus familiaris*): Genomic Inbreeding and Variability

**DOI:** 10.3390/ani10030498

**Published:** 2020-03-17

**Authors:** Antonio Boccardo, Stefano Paolo Marelli, Davide Pravettoni, Alessandro Bagnato, Giuseppe Achille Busca, Maria Giuseppina Strillacci

**Affiliations:** 1 Department of Veterinary Medicine, Università degli Studi di Milano, Via dell’Università 6, 26900 Lodi, Italystefano.marelli@unimi.it (S.P.M.); davide.pravettoni@unimi.it (D.P.);; 2 Centro Clinico-Veterinario e Zootecnico-Sperimentale, Università degli Studi di Milano, Via dell’Università 6, 26900 Lodi, Italy; giuseppe.busca@unimi.it

**Keywords:** run of homozygosity, dog, inbreeding, SNP, ROH, genetic diversity

## Abstract

**Simple Summary:**

In order to protect domestic animals’ biodiversity, a deep knowledge of the genomic makeup is required. The authors describe the genomic architecture of the German Short Hair Pointer breed and analyze the inbreeding levels under a genomic and a genealogic perspective. Twenty-four dogs from Italy were genotyped and analyzed jointly with 10 dogs from USA, whose genotypes were available from a published research. The authors investigated the genomic structural variation of the breed using runs of homozygosity—the direct measurement of the proportion of homozygous DNA, i.e., genomic inbreeding. Some traits clearly revealed the selection objectives addressed in the breed. The results describe a low inbred population with quite good levels of genetic variability.

**Abstract:**

The German Shorthaired Pointer (GSHP) is a breed worldwide known for its hunting versatility. Dogs of this breed are appreciated as valuable companions, effective trackers, field trailers and obedience athletes. The aim of the present work is to describe the genomic architecture of the GSHP breed and to analyze inbreeding levels under a genomic and a genealogic perspective. A total of 34 samples were collected (24 Italian, 10 USA), and the genomic and pedigree coefficients of inbreeding have been calculated. A total of 3183 runs of homozygosity (ROH) across all 34 dogs have been identified. The minimum and maximum number of Single Nucleotide Polymorphisms (SNPs) defining all ROH are 40 and 3060. The mean number of ROH for the sample was 93.6. ROH were found on all chromosomes. A total of 854 SNPs (TOP_SNPs) defined 11 ROH island regions (TOP_ROH), in which some gene already associated with behavioral and morphological canine traits was annotated. The proportion of averaged observed homozygotes estimated on total number of SNPs was 0.70. The genomic inbreeding coefficient based on ROH was 0.17. The mean inbreeding based on genealogical information resulted 0.023. The results describe a low inbred population with quite a good level of genetic variability.

## 1. Introduction

Dog selection is based on the breed standards that usually define the selection objectives for morphological traits and the behavioral predisposition of each breed [1]. Going back in human history, the domestication of dogs determined a genetic divergence from wild wolves, and the subsequent directional selection for specific characteristics led to a variety of dog breeds, counted to be around 500 [2]. Directional selection leads to a multitude of phenotypic variants for morphologic and behavioral traits [3,4].

Physical and physiological characteristics are determined by the genetic composition of the breeds which may have been affected by population bottlenecks due to specific directional selection practices. In this occurrence, a reduced number of ancestors, jointly to the large use of popular sires (e.g., the top winning male of the year) may result in an increase in homozygosity at population level [3,5,6]. 

The creation in the mid-19^th^ century of kennel clubs that defined breeds’ standards somehow formalized the reproductive isolation within the multiple canine morphologies present at that time [2]. A “breed-barrier rule” was then created, defining that every puppy may not enter a studbook unless its parents are registered in the same one [2]. The Kennel clubs’ studbooks activity in breeds preservation is based on strict pedigree control and evaluations of closeness to breeds standards. This process may lead to the fast reduction in the genetic variability within breeds, unless specific reproductive plans to control inbreeding are applied [7,8,9,10]. 

Previous studies have shown a decline in the genetic variability of some modern dog populations, characterized by high inbreeding levels like the Lundehund population where the pedigree-based inbreeding was 0.10 calculated on 11 generations [11,12,13,14,15]. The evaluation of genetic diversity represents a pivotal step for the establishment of a breeding program or to take conservation measures. An increase in homozygosity often leads to a loss in biological fitness due to a concomitant increase in the likelihood of the offspring being affected by deleterious or recessive traits [15]. Published studies report lower genetic diversity in breeds used for shows with respect to working dog breeds. These latter seem to maintain better genetic health due to the presence of many genetic traits still similar to those of their wild ancestors [13,16,17,18]. The characterization of genetic architecture, the study of inbreeding levels and the attention to the genetic health of dog in general should be considered the basis of effective selection plans, based on objective scientific information [16].

The traditional approach to inbreeding coefficient (IC) calculation is based on pedigree information: this value is clearly dependent on entries’ data accuracy and known generations in the pedigree (pedigree depth) [9,19]. The application of molecular genetics, and the construction of a high-density SNP maps [3] provide a powerful tool for the genomic IC calculation based on molecular information. Genomic IC can be measured via runs of homozygosity (ROH) (F_ROH_), representing a direct measurement of the fractions of homozygous stretches more likely to be transmitted identically by descent [9,10,16,18,19,20,21]. Another method is based on Wright’s F statistics, F_IS_ that returns the proportion of observed homozygosity respect the expected one [22]. The same powerful genomic tools can be used to provide molecular information to study the differentiation among breeds [20].

During domestication, hunting ability could have been considered among the most appreciated traits to select for; evidences have been found that 4000 years ago in the Middle East and North Africa, selective breeding for hunting aptitude was applied [23,24]. The typical hunting behavior of pointing breeds is represented by an interruption of the natural wolf predatory sequence, stopping in front of their prey [25,26]. Furthermore, the introduction of firearms in hunting drove a double goal in dogs’ performance ability: both pointing the pray and keeping away from the rifle [24].

The German Shorthaired Pointer (GSHP) breed represents the optimal evolution of pointing ability in German dog breeds. According to FCI standards, the history of the breed traces back to those dogs from Mediterranean countries that were used to hunt feathered game with nets and with raptors. Those dogs arrived in the German courts where they were selected primarily for their hunting ability and their versatility as gun dogs following the evolution of fire arms. The studbook of the breed, the “Zuchtbook Deutch-Kurzhaar” was firstly published in 1897. The standard of the breed—defining morphology and working characteristics—was written by Prince Albrecht zu Solms-Braunfeld. In Italy, the introduction of the breed began in the 1930s. GSHPs were exported to North America in the 1920s, and they entered the AKC studbook in 1930; recognized worldwide for their hunting versatility. Furthermore, they are appreciated as valuable companions, effective trackers, field trailers and obedience athletes. [27]. 

The aim of this study is to describe the genomic architecture of GSHP breed and to analyze the inbreeding levels of the breed under a genomic and a genealogic perspective, to supply effective data helpful for the design of breeding plans and conservation projects.

## 2. Materials and Methods 

### 2.1. Sampling and Genotyping 

The DNA of twenty-four pedigree GSHP samples (12 males, 12 female) was extracted from residual blood samples collected for routine screening of health status and according to the University Ethical Committee opinion n. 2/16 on re-use of collected samples. The samples were taken from unrelated, second-generation dogs from different Italian regions. This approach was aimed to make the sample here considered as much as possible representative of the Italian population and of its genetic variability. According to storage protocols, the dogs’ identities were certified by a veterinarian after the chip control: chip number, ENCI studbook registration number, name, sex and date of birth are the data commonly filled in the automated format used by the ENCI official labs in dog identification and recording procedures.

All dogs were genotyped with the Illumina CanineHD BeadChip array containing about 220,853 SNPs and the CanFam3.1 genome assembly (GCF_000002285.3) was considered as reference genome.

Ten genotyped German Shorthair Pointer dogs (GSHP_USA) from [8] (173662 SNPs - GSE90441 code project) were used in this study in order to increase the number of samples and to perform a better evaluation of selection targets related to breed morphology and pointing abilities. 

A final SNP dataset of 102,045 autosomal markers was obtained merging the two genotypes dataset: only SNP on autosomes were considered (from 1 to 38) and SNP genotypes data were filtered for minor allele frequency (MAF) (value ≤ 0.01) and for call rate (0.99), in order to reduce the bias ascribable to missing genotypes in inbreeding coefficients evaluation and in other statistics.

ADMIXTURE (v. 1.3.0) software [28] has been used to estimate the individual ancestries in the considered populations (the Italian GSHP_ITALY and the American GSHP_USA ones), and then to calculate the number of underlying possible subpopulations. ADMIXTURE was run from K = 1 to K = 4, and the optimal number of clusters (K-value) was determined as the one having the lowest cross-validation error (--cv flag added in command line). In addition, a Principal component analysis (PCA), based on the allele frequencies of SNPs, using SVS 8.4 software (SVS) (Golden Helix Inc., Bozeman, MT, USA) was performed.

### 2.2. Runs of homozygosity (ROH)

SVS software has been used to perform ROH detection. The ROH were defined setting a minimum of 1000 kb in size and 40 homozygous SNPs, no heterozygote SNPs are permitted, no missing SNPs were allowed in the ROH, and a maximum gap between SNPs of 1000 Kb was predefined in order to assure that the SNP density did not affect the ROH. 

ROHs were grouped into 5 classes of length (0-2 Mb, 2-4 Mb, 4-8 Mb, 8-16 Mb and, >16 Mb) and all the statistics were calculated across individuals within country (n = 24 for GSHP_ITALY and n = 10 for GSHP_USA) and for overall_GSHP (all 34 dogs).

The genomic regions with the highest frequency of ROH (TOP_ROH), and thus potentially under selection, have been identified by selecting the SNPs most commonly occurring in ROH, i.e., those occurring in at least 50% of samples plus one. The SNPs in the TOP_ROH are here referred to as TOP_SNPs. The TOP_SNPs and the TOP_ROH were annotated on CanFam3.1 genome (GCA_000002285.2, Release 99) using the Variant Effect Predictor (VEP) and the BioMart tools of Ensembl genome browser [29], respectively. Only genes with an official gene name were considered. Functional analysis of annotated genes has been performed using DAVID 6.8 database [30]. 

Graphical representations of ROH were obtained using the R package DetectRUNs [31].

### 2.3. Inbreeding Coefficients

In this study, genomic molecular inbreeding coefficients (FHOM and FROH) have been calculated for the overall_GSHP and the genealogical F for GSHP_ITALY (FPED) from pedigree information available only for Italian GSHP population:

(i) Inbreeding coefficients based on the excess in the observed number of homozygous genotypes (F_HOM_) with SVS, following [22]

F_HOM_ = (HomOb − HomEx)/(1 − HomEx) (1)

where HomOb and HomEx are the observed and expected numbers of homozygous genotypes in a dog. 

(ii) Inbreeding coefficient based on ROH (F_ROH_) with DetectRun, according to the following formula:

F_ROH_ = L_ROH_/L_aut_ (2)

where L_ROH_ is the total length of all ROHs of an individual, L_aut_ the specified length of the autosomal genome (from 1 to 38) covered by the SNPs used in this study (2,201,412,378 bp).

(iii) Inbreeding coefficient based on pedigree information (F_PED_) was calculated using Pedigree Viewer software [32]. Genealogical data of each sample dog (4 known generations) have been obtained using ENCI (Ente Nazionale della Cinofilia Italiana, FCI).

## 3. Results

**Figure 1:**
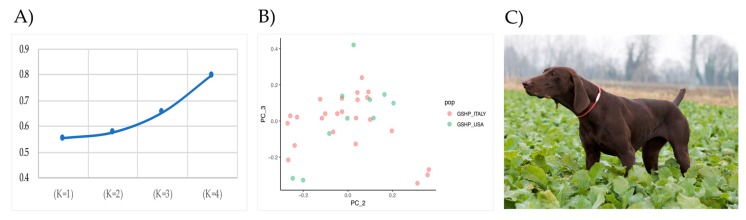
**A**) Cross-validation (CV) distribution calculated using in ADMIXTURE. Plot display CV errors versus K 1-4. **B**) PCA results and samples distribution (PC_2 = 0.67; PC_3 = 0.65). **C**) Example of GSHP dog (female).

**Figure 2:**
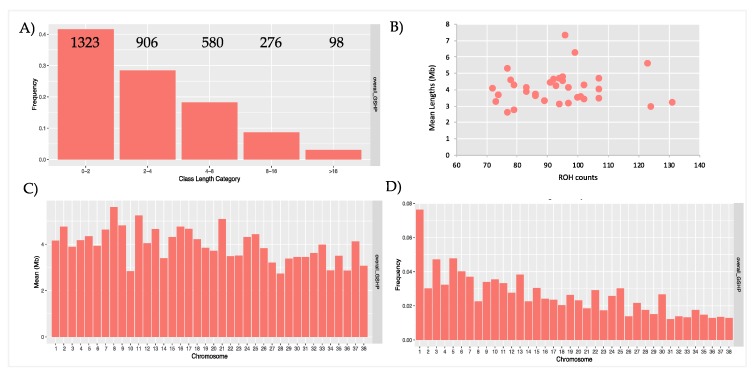
Graphical representation of runs of homozygosity (ROH) statistics. **A**) Frequencies and counts of ROH for each class of length; **B**) Relationship between number and averaged total length (Mb) of ROH in each dog; **C**) Mean length (Mb) for each chromosome; **D**) Frequencies of ROH per chromosomes.

ADMIXTURE analysis revealed that the GSHP_ITALY and GSHP_USA shared the same unique ancestor—as the lowest CV value has been obtained with K = 1 (Figure 1A)—and are considered then a unique breed. In addition, the PCA supported the results of the ADMIXTURE: as shown in Figure 1B, all individuals belong to a unique cluster. An example of a GSHP dog is shown in Figure 2C. 

All the results of this study are presented for overall_GSHP (n = 34 GSHP dogs). 

### 3.1. Runs of Homozygosity (ROH) 

The SVS software identified a total of 3183 runs across all 34 dogs (Table S1). Results revealed marked differences in terms of the number and length of ROH across individuals.

The minimum and maximum number of SNPs defining all ROH are 40 and 3060, respectively.

ROH are mainly short in length; in fact, the ROH of 0-2 Mb and 2–4 Mb are the most frequent classes of length identified (i.e., 70%) (Figure 2A). ROH were also found within the >16 Mb length class (Figure 2A).

The number of ROH per individual ranged from 71 to 131, with a mean number of ROH for sample of 93.6 (Table 1). Only three samples showed a very large number of ROH, with counts of 123, 124 and 131. The average size of the ROH of these three individuals is nevertheless similar to the one of the other dogs (5551411 bp, 2,902,950 bp and 3,203,416 bp). Figure 2B shows the relationship between ROH count and the average total length of ROH for each individual (mainly ranged 3 to 5 Mb). The amount of the genome covered by ROH per dog ranged (as mean values) from 2,557,174 bp to 7,312,103 bp.

ROH were found on all chromosomes: less than 50% of chromosomes (n.17) (Figure 2C) have a mean length over 4.072 Mb (mean ROH length, as reported in Table 1), and no evident correlation between chromosomes length and mean ROH length resulted. A graphical representation of ROH frequencies on autosomes is shown in Figure 2D. The number of ROH within each class of length per chromosome was also calculated (Table S2): the longest ROH (>16 Mb) were identified on almost all the chromosomes, except for chr23, chr27, chr32, chr33, chr34, and chr36. A graphical representation of ROH statistics for Italian and USA populations are shown separately in Figure S1 A–E.

**Figure 3:**
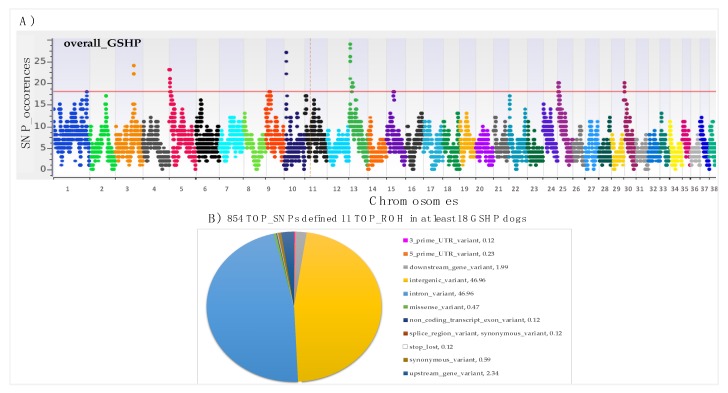
SNPs occurrences in identified ROH and their annotation on canine genome assembly CanFam3.1: **A**) Manhattan plot: red line indicates the adopted threshold: 50% of samples + 1; **B**) Annotated position of TOP_SNPs: numbers in legend of graph represent percentages of each identified position respect to a gene.

The Figure 3A shows the SNP occurrences in ROH segments across the genome, highlighting also that the genomic distribution of ROH is clearly non-uniform across autosomes. A total of 11 TOP_ROH on 9 chromosomes were identified in at least 18 samples (50% of sampled dogs +1) (red line in Figure 3A) (Table 2). The higher chromosomal peaks were identified on CFA 3, 10 and 13. Table 2 also reports the number of samples in which the TOP_ROH have been identified (min = 18 and max = 29), the list of genes (n.116) present in the identified TOP_ROH, and the list of genes (n. 15) for which an already known association with canine traits has been described (Table 2 – in bold, and detailed in Table S6).

We found a total of 854 TOP_SNPs, and Figure 3B is the graphical representation of the annotated position of these SNPs (11 classes of position) according to Ensembl VEP. The major part of SNPs mapped in intergenic (n. 401; 46,96%) and in intronic positions (n. 401; 46,96%) (Figure 3B). Details of all the TOP_SNPs positions and annotation information are reported in Table S4. 

The highest number of homozygotes SNPs (> 25) was annotated within the *FMN1* (n. 26), *RYR3* (n. 28), *CSMD3* (n.29), *OPCML* (n. 33), and *NTM* (n. 50) genes. In addition, four SNPs resulted annotated in missense positions: BICF2P1347925, BICF2S23147347 and TIGRP2P48933_rs8545710 on chr3 and BICF2P847459 on chr30 of *HTT*, *HTT*, *ADD1*, and *RYR3* genes, respectively. SNP genotypes of the dogs for each of these positions were verified and, as reported in Table S3, resulted in: all wildtype genotype for BICF2P1347925, TIGRP2P48933_rs8545710 and BICF2P847459 (n. 22, 22 and 20, respectively) with AA variant coded as AA genotype; all mutated variants were found for BICF2S23147347 (n. 22) with variant CC coded as BB genotype. Table S3 also includes genotypes for these missense positions for the dogs that did not contribute to these ROH.

Functional classification of genes annotated in TOP_ROH (Table 3) provided by DAVID database (112 gene IDs recognized) revealed that these genes were significantly enriched (nominal p-value <0.05) in three GO terms in biological processes, only one GO term in cellular components and seven KEGG pathways, for which the *CACNG5, CACNG4, CACNG1* are the main involved genes (Table 3). 

Table S5 reports the GO terms and KEGG pathways resulted with nominal *p*-value >0.05.

### 3.2. Inbreeding Coefficients

The average observed and expected homozygotes calculated using 102,045 SNPs were 71785.44 and 70513.85, respectively. The proportion of average observed and expected homozygotes estimated on the total number of SNPs were then 0.70 and 0.69. 

The inbreeding coefficient estimated from SNP markers (F_HOM_, equation 1) was 0.04 (averaged values) and individual values ranged from −0.06 to 0.21 for dogs with lower than average homozygosity and vice versa, respectively. 

Instead, the inbreeding coefficients based on ROH (F_ROH_, equation 2) were slightly higher: a mean value of 0.17, ranging between 0.09 to 0.32. Differences in F_ROH_ were found along all chromosomes (Figure S2). The higher F_ROH_ values (≥ 0.25) have been identified for chrs 9, 13 and 25. 

**Figure 4:**
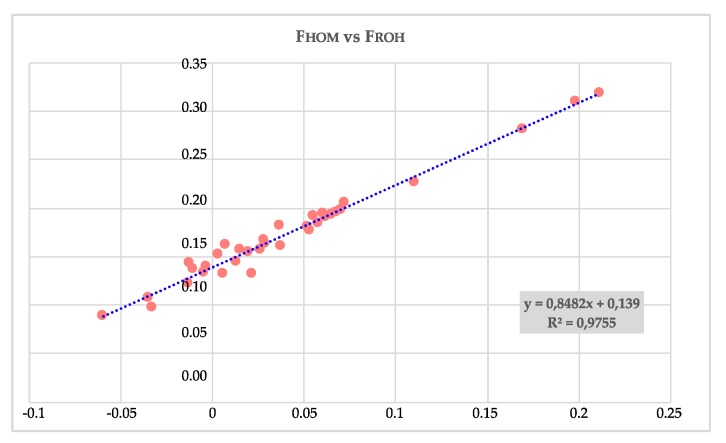
Regression and coefficient of determination (R^2^) calculated between F_HOM_ and F_ROH:_ y = F_ROH_; x = F_HOM._

The correlation coefficient, and that of determination (R ^2^) calculated between F_HOM_ and F_ROH,_ were 0.988 and 0.975 (as reported in Figure 4), respectively.

The four-generation pedigree analysis resulted in a very low F_PED_ coefficient for all the samples: 14 subjects showed no genealogical inbreeding, only four GSHPs revealed a F_PED_ higher than 0.05, the maximum F_PED_ was 0.161 (Table S6). The mean F_PED_ of the studied population was 0.023 with a standard deviation of 0.043. The regression between F_HOM_ and F_PED_ (Figure S3A) showed a similar relationship to and F_PED_ and F_ROH_, with a regression coefficient of 0.83 vs. 0.84, but with a lower coefficient of determination (R ^2^ of 0.427 vs. 0.97). Figure S3B shows the regression between F_ROH_ and F_PED_, resulting in a lower coefficient but similar R^2^ to F_HOM_ and F_PED_.

## 4. Discussion

Not many studies have explored the ROH pattern and its possible association with inbreeding depression, to morphological and health traits in dog breeds and populations in comparison to researches performed and available in other species (i.e., cattle). The ROH patterns and their distributions in specific dog breeds remain largely unexplored.

To the best of our knowledge, this study is the first attempt to characterize the ROH distribution in the German Shorthair Point dog breed using canine high-density SNP arrays. The genealogical pedigree information was used to sample the 24 individuals that were as un-related as possible. Ten available online genotyped GSHP samples from the GSPH_USA populations were available from a previous published study [8] and added to the 24 Italian dogs in order to increase the sample size of this study. According to the results of ADMIXTURE and the PCA which hereinbefore presented the detection of ROH are here presented jointly. Details of the ROH for GSPH_USA and GSPH_Italy are shown in Figure S1 A–E. The results obtained allow us to disclose the variability in the GSPH breed and to identify the genomic region harboring ROH, even if enlarging the sample size in future efforts may disclose additional information on genomic variation in this population. 

The detection of genomic regions (i.e., TOP_ROH) underlying breed-specific phenotypic characteristics or attitude, can provide indication about a specific genetic structure of a population or breed. ROH, in concordance with their length, inform on a possible artificial or natural selection pressure on specific genomic tracts and on inbreeding levels also providing information on bottlenecks to which the populations have been subjected over the years. Long ROH (~ 10 Mb) occur as a result of recent inbreeding and when recombination events do not interrupt long chromosome segments. Short ROH (~ 1 Mb), instead, are produced by IBD (identical by descent) genomic regions from old ancestors (up to 50 generations ago) [33].

Similar to what has been identified in some hunting dog breeds—the French Pointing dog type the Pyrenee and in Rhodesian Ridgeback dogs, respectively—most of the ROH identified in this study were short in length (0-2 Mb, with a mean length of 1.42 Mb), suggesting that dogs of these populations are involved in more ancient relatedness [18,34]. The occurrence of TOP_ROH hotspots in genomic regions that harbor candidate genes may be involved in directional selection pressure. Out of 854 TOP_SNPs, 452 are annotated within genes according with VEP tool (taking into account different positions, as in Figure 3A). The most representative genes (those in which at least 25 TOP_SNPs map) resulted *NTM, OPCML, CSMD3, RYR3, FMN1, TMTC2,* and *HTT*. All these genes (except for *NTM*), together with others (n. 15) have been already associated with dog traits, comprising behavior and morphological traits, as reported in Table 4.

*NTM* and *OPCML* (the most representative) are genes involved in central nervous system functioning. Studies performed in humans proposed that the *NTM* gene (Neurotrimin) is associated with IQ level and cognitive function performances. In addition, Gurgul et al. [37] supposed in their study on diversifying selection signature between draft and light horses, the potential contributes of *MNT* and *OPCML* (Opioid Binding Protein/Cell Adhesion Molecule Like) genes to the differing horses’ temperaments and to the ability to develop different gaits as a function of motor coordination. It could be supposed that these genes’ expression, considering the presence of many TOP_SNPs, could be related to the peculiar hunting abilities and style of GSHPs. These traits have been strongly selected by the breeders since the 19^th^ century, and they are clearly described in the breed working standards [26,35]. *RYR3* (Ryanodine Receptor 3), together with *GRK4* (G protein-coupled receptor kinase 4) located in TOP_ROH_02, resulted genes positively selected in athletic dog breeds (sports and hunting aptitudes). In fact, these two genes could have an important role in field activity: *RYR3* because of its function in the activation of muscle skeletal contraction through the coordinate activation of voltage dependent Ca2+ [38], and *GRK4* in increasing cardiac output. 

The only gene for which an SNP missense position (BICF2S23147347) with alternative variant G (coded as B allele) has been found (Table S4), according to VEP tool, is the *HTT* gene. No specific association study results are yet available for this mutation or gene in the dog species. The only exception is for chasing behavior trait, as reported in Table 4.

Another gene that we could hypothesize under selection in this breed, according to breed-specific characteristics described in the standard, is that the encoding factors involved in skeletal muscle contraction processes—i.e., *CACNG1* (calcium voltage-gated channel auxiliary subunit gamma 1) —: a polymorphism of *CACNG1* gene has been associated with elite strength athlete status [39]. *CACNG1*, *CACNG4* (subunit gamma 4)*,* and *CACNG5* (subunit gamma 5) are genes implicated in five KEGG pathways related to cardiac functions (contraction and healthy). These three genes are also part of the MAPK signaling pathway, active on the proliferation of mammalian cells, and play an important role at different levels—e.g., the regulation of various diseases (cardiovascular), taking part in anti-inflammatory effects, responses to stress, protection against injury, and the maintenance of gastrointestinal functions [40].

The loss of genetic variability and the increase in inbreeding levels coupled with the consequent reduction in the effective population size in a high number of pedigree canine populations could be considered the main risk factor in conservation project definitions [12,15,41,42,43,44]. In general, the reported results show low F_PED_ in the studied subjects [44]. The effectiveness of the molecular dissection of inbreeding is shown by the F_ROH_ (equation 2) results, which reveal the genomic relationship among the studied individuals as demonstrated by different researchers [8,16,45]. The proportion between observed and expected homozygosity reveals similar results to those reported by [18] about the French Pointing dog Type Pyrenee (1.03 vs. 1.02). The obtained inbreeding coefficient F_HOM_ (equation 1) based on SNPs is lower compared to all the results reported in 11 breeds by [8], which ranged from 0.179 in the Papillon to 0.53 in the Basenji. When considering the inbreeding coefficient based on ROH the obtained results are the same of those presented by [34] with a registered level of 0.17. The same coefficient calculated in the French Pointing dog Type Pyrenee was slightly lower: 0.112 [18]. Considering pedigree data analysis, the obtained results in the studied population are slightly lower when compared to other pointing breeds registered in the Italian studbook (ENCI): in the Italian Pointing Dog, the pedigree average inbreeding coefficient was 0.041 [46], and in the French Pointing Dog type Pyrenees F was calculated to be 0.033 [47]—in both cases, the whole registered population was considered. The results we obtained on a sample population are close to those reported by [8], who compared pedigree average genealogical inbreeding with SNP chip and whole genome sequence (WGS) heterozygosity: the closeness of our results it is not only related to the pedigree (five generations) inbreeding coefficient of very famous breeds known worldwide like the Labrador Retriever (0.026), Golden Retriever (0.027) and Bernese Mountain Dog (0.022), but the same differences reported comparing pedigree inbreeding with SNPs and WGS inbreeding levels has been described in the Italian GSHP sample population. Inbreeding levels considering pedigree data were considerably lower than the average inbreeding levels obtained by molecular genomic data analysis.

## 5. Conclusions

The investigation of the genomic architecture and the molecular dissection of inbreeding play a pivotal role in the conservation and protection plans for animal populations. Furthermore, genomic analysis supplies effective tools with high quality molecular data in the demographic profiling of canine populations. The presented results represent the first description of the molecular dissection of the inbreeding levels in the Italian and USA pointing GSHP breeds. The genomic molecular investigation allowed the authors to finely describe the genetic assets of the breed, even if enlarging the sample size may disclose other regions under recent inbreeding. The obtained results could be considered a powerful and objective tool in breed conservation management. The genomic data here analysed showed important homozygosity for morphological and behavioural traits, whose expression is contributed by known annotated genes. The findings of the research also underline the importance of integrating genealogical and molecular information on dog breeds, in order to obtain meaningful data for breeds’ protection actions.

## Figures and Tables

**Table 1:** Descriptive statistics of ROHs.

POP	N. ROHs	Min - Max (Mean) of ROH Per Samples	Min ROH Length	Max ROH Length	Mean ROH Length	(%) Total Coverage
overall_GSHP	3183	71-131 (93.6)	1000717	56893025	4072407	5.9

**Table 2:** TOP_ROHs found in at least 50% of overall_GSHP samples +1 (n = 18) and annotated genes.

TOP_ROH_id	Chr	Start Position	End Position	*N. Samples	Genes
TOP_ROH_1	1	112009648	112324183	18	CNFN, MEGF8, TMEM145, PRR19, PAFAH1B3, CIC, ERF, GSK3A, ZNF526, DEDD2, POU2F2, ZNF574, GRIK5
TOP_ROH_2	3	60892911	62824148	22-24	DOK7, HGFAC, RGS12, MSANTD1, **HTT**, GRK4, NOP14, MFSD10, ADD1, SH3BP2, TNIP2, **FAM193A**, **RNF4**, CFAP99, ZFYVE28, MXD4, HAUS3, *POLN, NAT8L, C3H4orf48, NELFA, SCARNA22, NSD2, LETM1, FGFR3, TACC3, TMEM129, SLBP, FAM53A, NKX1-1, UVSSA, MAEA, FAM184B*
TOP_ROH_3	5	247752	3480976	18-23	B3GAT1, GLB1L2 (LOC606786), GLB1L3, ACAD8, THYN1, cfa-mir-8868, VPS26B, NCAPD3, JAM3, IGSF9B, **SPATA19**, **OPCML**, NTM
TOP_ROH_4	5	4900070	4979148	18	PRDM10, NFRKB
TOP_ROH_5	9	12539669	14668121	18	KPNA2, C9H17orf58, BPTF, **NOL11**, PITPNC1, PSMD12, HELZ, CACNG1, CACNG4, CACNG5, PRKCA, APOH, CEP112, AXIN2
TOP_ROH_6	10	7278956	8418771	22-27	*RASSF3, GNS, **TBC1D30**, **WIF1**, LEMD3, **MSRB3**, **HMGA2***
TOP_ROH_7	13	2962719	4505605	21-29	ZNF706, *GRHL2, **NCALD**, RRM2B, UBR5, ODF1, KLF10*, AZIN1
TOP_ROH_8	13	10151357	13036989	18-20	SYBU, KCNV1, **CSMD3**
TOP_ROH_9	15	24204830	25089306	18	CCDC59, METTL25, **TMTC2**
TOP_ROH_10	25	2268042	3887386	18-20	UFM1, TRPC4, POSTN, SUPT20H, EXOSC8, ALG5, SMAD9, RFXAP
TOP_ROH_11	30	927947	2775667	18-20	EMC7, CHRM5, AVEN, **RYR3**, **FMN1**, GREM1, SCG5, ARHGAP11A, GJD2, ACTC1, AQR, ZNF770

(*) = min and max number of samples with TOP_ROH; in *Italics* = genes in which map the highest number of TOP_SNPs; in **bold** = genes already associated with canine traits.

**Table 3:** Functional classification of genes according with DAVID database: BP = Biological Process, CC = Cellular Component: KEGG = KEGG pathways.

Category	Term (Nominal *p*-Value)	Genes
	GO:0019226~transmission of nerve impulse (0.005)	CHRM5, CACNG5, JAM3
GOTERM (BP)	GO:0071407~cellular response to organic cyclic compound (0.02)	SMAD9, GSK3A, AXIN2
	GO:0043161~proteasome-mediated ubiquitin-dependent protein catabolic process (0.035)	MAEA, RNF4, PSMD12, GSK3A
GOTERM (CC)	GO:0005654~nucleoplasm (0.01)	HAUS3, SMAD9, HTT, NFRKB, RRM2B, GRHL2, BPTF, UBR5, SYBU, POLN, PITPNC1, AXIN2, TNIP2, KPNA2, DEDD2, ADD1
KEGG	cfa04260: Cardiac muscle contraction (0.005); cfa05410: Hypertrophic cardiomyopathy (HCM) (0.007); cfa05414: Dilated cardiomyopathy (0.008); cfa04261: Adrenergic signaling in cardiomyocytes (0.028).	ACTC1, CACNG5, CACNG4, CACNG1
	cfa04921: Oxytocin signaling pathway (0.005); cfa04010: MAPK signaling pathway (0.032).	PRKCA, RYR3, CACNG5, CACNG4, CACNG1
	cfa05412: Arrhythmogenic right ventricular cardiomyopathy (ARVC) (0.042)	CACNG5, CACNG4, CACNG1

**Table 4:** Genes already associated with canine traits in the scientific literature.

TOP_ROH_id	Gene Name	Behavior Traits [35]	Morphological Traits [36]
TOP_ROH_2	*FAM193A*	Chasing, Dog Aggression	
TOP_ROH_2	*HTT*	Chasing	
TOP_ROH_2	*RNF4*	Chasing, Dog Aggression	
TOP_ROH_3	*NCAPD3*	Separation Problems	
TOP_ROH_3	*SPATA19*	Chasing	
TOP_ROH_5	*NOL11*	Energy	
TOP_ROH_5	*OPCML*	Dog Aggression	
TOP_ROH_6	*HMGA2*		Body size
TOP_ROH_6	*MSRB3*	Dog Fear	Ear morphology
TOP_ROH_6	*TBC1D30*	Attachment/attention-seeking, Dog Fear	
TOP_ROH_6	*WIF1*		Ear morphology
TOP_ROH_8	*CSMD3*	Excitability	
TOP_ROH_9	*TMTC2*	Attachment/attention-seeking, Excitability	
TOP_ROH_11	*FMN1*	Energy, Trainability	
TOP_ROH_11	*RYR3*	attachment/attention-seeking, Energy	

## Supplementary Materials

The following are available online at https://www.mdpi.com/2076-2615/10/3/498/s1, Figure S1: Graphical representation of ROH statistics for Italian and USA GSHP. Figure S2: Graphical representation of F_ROH_ value for each chromosome. Figure S3A: Regression and coefficient of determination (R^2^) calculated between F_HOM_ (Y) and F_PED_ (X); Figure S3B: Regression and coefficient of determination (R^2^) calculated between F_ROH_ (Y) and F_PED_ (X). Table S1: List of ROH identified in GSHP dog breed. Table S2: ROH count per chromosomes and per classes of ROH. Table S3: TOP_SNPs annotation. Table S4: Samples genotypes per TOP_SNPs missense positions. Table S5: Functional annotation of genes mapping in TOP_ROH according with DAVID database (nominal *p*-value >0.05).
