# Supplementary figures and images for: The German Shorthair Pointer Dog Breed (Canis lupus familiaris): Genomic Inbreeding and Variability
Source: Animals (Basel). 2020 Mar 17;10(3):498. doi: 10.3390/ani10030498 (PMC7143860; doi:10.3390/ani10030498)

A) FHOM vs FPED

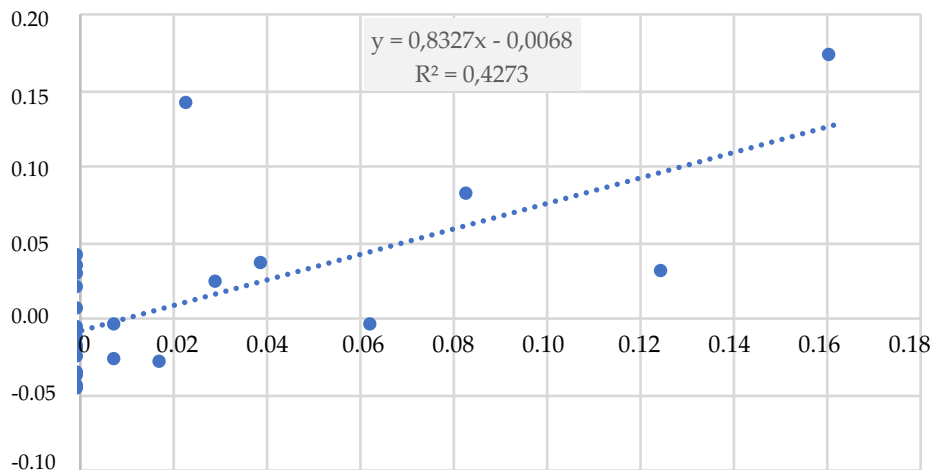

B) FROH vs FPED

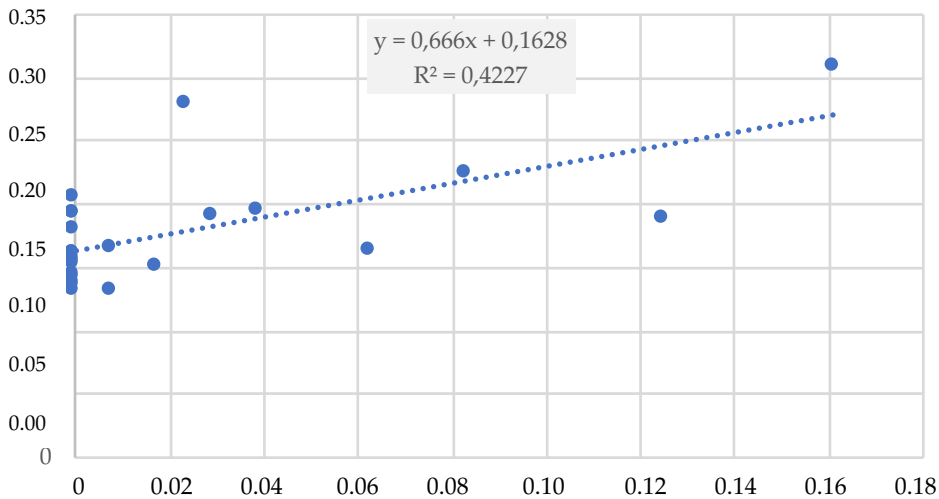

Supplement: Supplementary file 1 [file animals-10-00498-s001.zip › Additional_Files/Figure_S3.pdf]

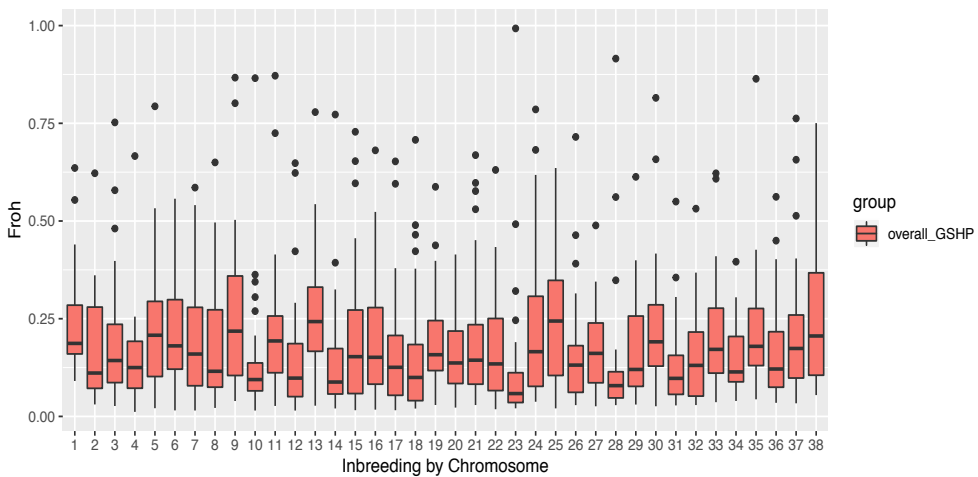

Supplement: Supplementary file 1 [file animals-10-00498-s001.zip › Additional_Files/Figure_S2.pdf]

A)

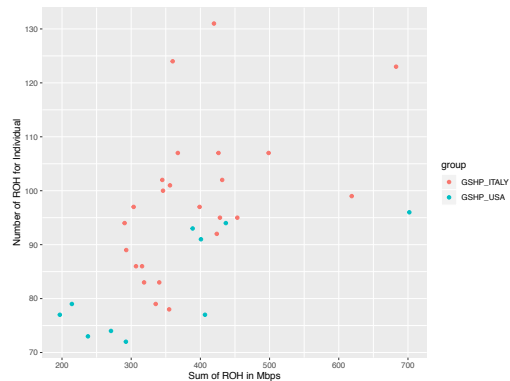

B)

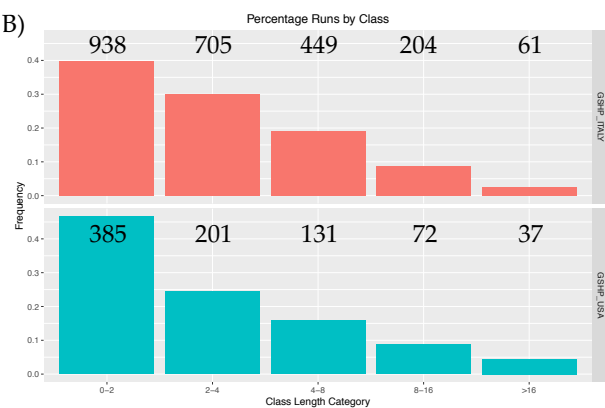

C)

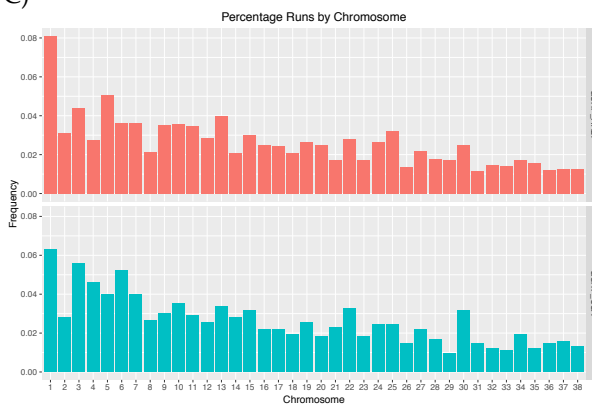

D)

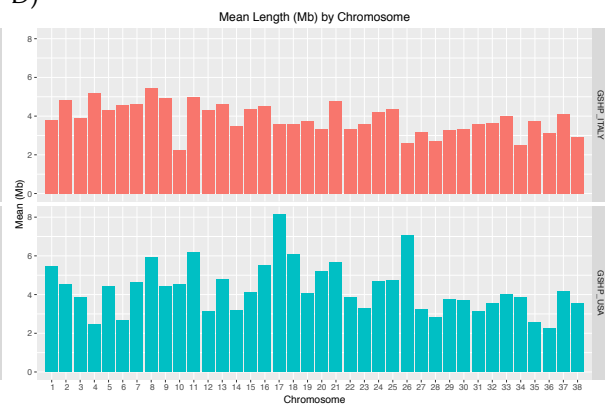

E)

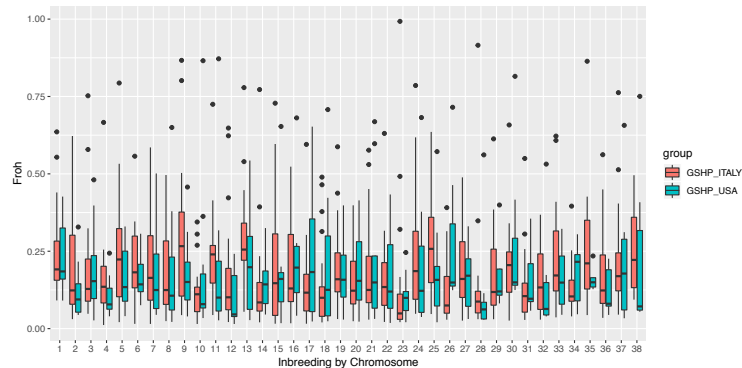

Supplement: Supplementary file 1 [file animals-10-00498-s001.zip › Additional_Files/Figure_S1.pdf]
